# Biventricular Remodeling in Murine Models of Right Ventricular Pressure Overload

**DOI:** 10.1371/journal.pone.0070802

**Published:** 2013-07-30

**Authors:** Navin K. Kapur, Vikram Paruchuri, Mark J. Aronovitz, Xiaoying Qiao, Emily E. Mackey, Gerard H. Daly, Kishan Ughreja, Jonathan Levine, Robert Blanton, Nicholas S. Hill, Richard H. Karas

**Affiliations:** Molecular Cardiology Research Institute, Tufts University School of Medicine, Boston, Massachusetts, United States of America; Albert Einstein College of Medicine, United States of America

## Abstract

Right ventricular (RV) failure is a major cause of mortality in acute or chronic lung disease and left heart failure. The objective of this study was to demonstrate a percutaneous approach to study biventricular hemodynamics in murine models of primary and secondary RV pressure overload (RVPO) and further explore biventricular expression of two key proteins that regulate cardiac remodeling: calcineurin and transforming growth factor beta 1 (TGFβ1).

**Methods:**

Adult, male mice underwent constriction of the pulmonary artery or thoracic aorta as models of primary and secondary RVPO, respectively. Conductance catheterization was performed followed by tissue analysis for changes in myocyte hypertrophy and fibrosis.

**Results:**

Both primary and secondary RVPO decreased biventricular stroke work however RV instantaneous peak pressure (dP/dt_max_) and end-systolic elastance (Ees) were preserved in both groups compared to controls. In contrast, left ventricular (LV) dP/dt_max_ and LV-Ees were unchanged by primary, but reduced in the secondary RVPO group. The ratio of RV:LV ventriculo-arterial coupling was increased in primary and reduced in secondary RVPO. Primary and secondary RVPO increased RV mass, while LV mass decreased in primary and increased in the secondary RVPO groups. RV fibrosis and hypertrophy were increased in both groups, while LV fibrosis and hypertrophy were increased in secondary RVPO only. RV calcineurin expression was increased in both groups, while LV expression increased in secondary RVPO only. Biventricular TGFβ1 expression was increased in both groups.

**Conclusion:**

These data identify distinct effects of primary and secondary RVPO on biventricular structure, function, and expression of key remodeling pathways.

## Introduction

Right ventricular (RV) failure is a major determinant of morbidity and mortality for millions of individuals worldwide who suffer from pulmonary hypertension (PH) due to acute and chronic lung disease, or left heart failure [1]–[3]. Several studies have confirmed that elevated pulmonary artery systolic pressures are inversely associated with RV systolic function in both primary and secondary PH [4], [5]. However, the fundamental mechanisms underlying the development of RV failure in these populations remain poorly understood.

Ventriculo-arterial coupling describes the impact of arterial loading conditions on ventricular function. Under any given condition, optimal pump efficiency is achieved if ventricular function, or end-systolic elastance (Ees), is matched by vascular load, known as arterial elastance (Ea) [6]–[10]. Since the majority of RV stroke work maintains forward momentum of blood flow into a compliant, low resistance circulation, small increases in afterload can reduce RV stroke volume [11]. Under conditions of RV pressure overload (RVPO) within an intact pericardium, reduced RV output will impact left ventricular (LV) function by decreasing LV preload, increasing coronary sinus pressure, and mechanically impinging on the LV throughout the cardiac cycle [12]–[13]. While biventricular interactions have been the subject of intensive study in left heart failure, few studies in RVPO have focused on RV remodeling [14]–[17]. The impact of RVPO on biventricular function remains poorly characterized.

As the thin-walled RV dilates in the setting of pressure overload, increased RV wall stress activates signaling cascades that promote cardiac hypertrophy and fibrosis including the transforming growth factor beta-1 (TGFβ1) and calcineurin pathways. TGFβ1 is a powerful pro-fibrogenic cytokine that signals through a heteromeric receptor complex comprised of a Type II ligand-binding receptor, Type I activin like kinase (ALK) signaling receptors, and the Type III accessory co-receptor, Endoglin. Upon TGFβ1 activation, this receptor complex phosphorylates downstream effector proteins known as Smads (canonical pathway) or mitogen activated protein kinases, like extracellular regulated kinase (ERK; non-canonical pathway) [18]–[20]. Specifically, TGFβ1-induced phosphorylation of Smad-2/3 and ERK promotes Type I collagen synthesis and fibrosis. Given its central role in stimulating fibrosis, TGFβ1 has been non-selectively targeted in models of left heart failure, using multiple approaches; none of which have produced clearly beneficial therapeutic effects [21], [22]. TGFβ1 signaling in RV remodeling has remained largely ignored. Models of left heart failure have also confirmed the central role of the calcium-dependent serine/threonine phosphatase, calcineurin, as a mediator of maladaptive hypertrophy secondary to pressure overload. Calcineurin dephosphorylates nuclear factor of activated T cells (NFAT), a family of cytoplasmic transcription factors that promote gene expression that regulates cardiac hypertrophy [23], [24]. Recent studies have also implicated calcineurin as a key regulator of cardiac fibrosis [25]. Few studies have examined the impact of these signaling pathways in RV and LV remodeling in the setting of RVPO [26].

Given the increasing use of murine models of left heart failure and PH, the objective of this study was to demonstrate a novel percutaneous approach to study biventricular hemodynamics in clinically relevant murine models of primary and secondary RVPO and further explore biventricular expression of two key proteins that regulate cardiac remodeling: calcineurin and TGFβ1.

## Methods

### Murine Models of Right Ventricular Pressure Overload

Animals were treated in compliance with the Guide for the Care and Use of Laboratory Animals (National Academy of Science), and protocols were approved by the Tufts Medical Center Institutional Animal Care and Use Committee. Adult, 12–14 week-old male C57/Bl6 mice (n = 12/group) underwent constriction of the pulmonary artery or thoracic aorta as previously described to generate models of acute primary and progressive secondary RVPO respectively [14], [19]. Briefly, mice were intubated using a 24G angiocath and mechanically ventilated (Harvard Apparatus) at 95 breaths per minute with a tidal volume of 0.3 mL with 2.0–2.5% Isoflurane and 100% flow-through oxygen. Depth of anesthesia was monitored by assessing palpebral reflex, toe pinch, respirations, and general response to touch. Using sterile technique, a left thoracotomy was performed to isolate and encircle the main pulmonary artery or transverse thoracic aorta using a 7–0 nylon suture that is then tied tightly around a pre-sterilized, blunt end 27G needle for pulmonary artery or thoracic aortic constriction. After de-airing, the thorax is closed with layered 6-0 Dexon sutures to minimize the risk of pneumothorax. Post-operative analgesia is immediately provided with Buprenorphine 0.1 mL, which is continued twice daily and as needed for an additional 72 hours. Mice were allowed to survive for 7 days or 3 weeks after pulmonary artery constriction (PAC) and 7 days or 10 weeks after thoracic aortic constriction (TAC). Two groups of controls (n = 6/group) were studied 7 days and 10 weeks after sham surgery.

### Biventricular Conductance Catheter Instrumentation

**Figure 1:**
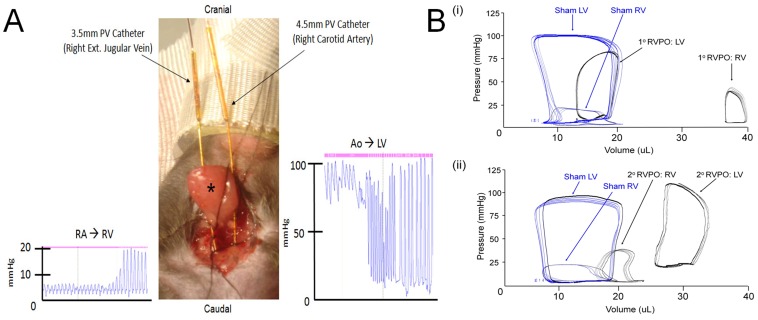
Biventricular conductance catheterization in a closed-chest, non-invasively ventilated mouse. A) Hemodynamic tracings illustrating pressure volume (PV) catheters tracking from the right atrium (RA) to right ventricle (RV) via the right external jugular vein and aorta (Ao) to left ventricle (LV) via the right carotid artery via a suprasternal incision in a closed-chest mouse (*, salivary gland). B) Representative steady-state PV loops in mouse models of (i) primary and (ii) secondary right ventricular pressure overload (RVPO) (blue loops represent sham operated animals).

All animals underwent terminal hemodynamic evaluation. Biventricular catheterization was performed at the time of sacrifice in all animals. Mice were anesthetized with 2.0% isoflurane administered via a non-invasive nose-cone. Body temperature was monitored by a rectal thermistor probe and maintained at 37.5°C with heating pads and a cycling heat lamp. In the supine position, the right common carotid and right external jugular vein were surgically isolated. Silk ties were placed at the distal ends of both vessels while overhand loops were placed at the proximal ends with 7-0 nylon. Millar PVR-1035 and PVR-1045 (Millar Instruments, Inc.; Houston, Texas) mouse conductance catheters and independent consoles were used for right and left ventricular recordings respectively. Prior to insertion, conductance catheter calibration was performed using the cuvette method with freshly heparinized warm blood as previously described [15]. Both catheters were then zeroed in warm saline. A transverse venotomy was performed using iris scissors at the proximal end of the external jugular vein. The PVR-1035 catheter was advanced through the SVC and right atrium into the right ventricle. For LV cannulation, a micro vascular clip was first placed proximal to the nylon overhand loop, then a transverse arteriotomy was performed with iris scissors and the PVR-1045 catheter was advanced to the clip. The proximal nylon was then tightened around the vessel and the catheter. After removing the surgical clip, the catheter was maneuvered past the aortic valve into the LV (Figure 1A).

### Hemodynamic Study Protocol

For the primary (7 day PAC; n = 6/12) and secondary (10 week TAC; n = 6/12) RVPO groups, mortality was 50%, therefore 6 mice/group underwent analysis. All mice (n = 4/4) survived in the 7 day secondary RVPO (TAC) group and underwent analysis. Mortality approached 85% (n = 10/12) in the 3 week primary RVPO (PAC) group, which precluded further analysis. Once hemodynamic stability was achieved, steady-state baseline conditions were recorded from the RV first. To minimize interference due to local electric field distributions from two catheters in close proximity, the console for the RV conductance catheter was paused and steady-state baseline conditions were immediately recorded from the LV conductance catheter and console. The RV catheter was then re-activated and data was acquired sequentially from the RV, then LV during occlusion of the inferior vena cava (IVC). For IVC occlusion, a small incision inferior to the xyphoid was made and blunt dissection was used to visualize the IVC. Transient occlusion of the IVC was performed with a micro-vascular clip. Using the multiple beat method with variable preload, end-systolic elastance (Ees) was defined as P(t)[V(t)-V0], where P(t) is instantaneous pressure, V(t) is instantaneous volume, and V_0_ is a theoretical estimate of volume at zero pressure [27]. Arterial elastance (Ea) was calculated under steady-state conditions as end-systolic pressure/stroke volume. Ejection fraction was calculated as stroke volume divided by end-diastolic volume. PV loop acquisition and analysis was performed using IOX software (EMKA). After completion of the hemodynamic study, with the animal still under isoflurane anesthesia, the chest was rapidly opened, and the mouse was euthanized by arresting the heart in diastole with 0.3 mL of 1 N KCL injected directly into the left ventricle. The heart was then removed and processed for either biochemical or histologic analyses. All surgical procedures and tissue harvesting were performed in concordance with the National Institutes of Health and had approval of the Institutional Animal Care and Use Committee (IACUC) at Tufts Medical Center and the Tufts University School of Medicine.

### Histologic Quantification of Cardiac Hypertrophy and Fibrosis

RV and LV collagen abundance by picrosirius red staining were quantified as percent fibrosis of the total RV and LV respectively. Cardiomyocyte cross-sectional area was quantified as previously described [28]–[30].

### Real-time Quantitative Polymerase Chain Reaction (RT-PCR)

For RT-PCR, total RNA was extracted from RV and LV tissues directly using Trizol (Invitrogen), converted to cDNA using a High Capacity cDNA Reverse Transcription Kit (Applied Biosystems). For all RT-PCR experiments, samples were quantified in triplicate using 40 cycles performed at 94°C for 30 sec., 60°C for 45 sec, 72°C for 45 sec using an ABI Prism® 7900 Sequence Detection System using appropriate primers as previously described [28]–[30].

### Immunoblot Analysis (Western)

Total protein was extracted and quantified from tissue homogenates as described (28–30). Immunoblot analysis was then performed as previously described using antibodies for mouse targeted proteins including: Type I collagen (Santa Cruz Inc), calcineurin (Cell Signaling), pERK (Millipore), total ERK (Cell Signaling), pSmad-3 (Santa Cruz Inc), and total Smad-3 (Cell Signaling).

### Statistical Analysis

Results are presented as mean ± standard deviation. Intergroup comparisons were made with a Student’s t-test and two-factor ANOVA. All statistical analyses were performed using SigmaStat Version 3.1 (Systat Software, Inc). An alpha level of P<0.05 was considered to indicate a significant effect or between-groups difference.

## Results

### Biventricular Hemodynamics in RVPO

**Figure 2:**
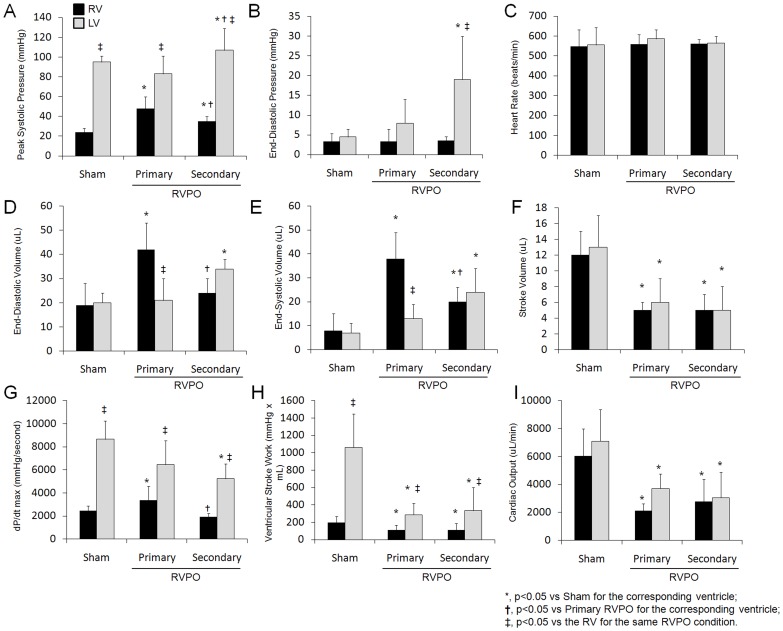
Biventricular hemodynamics in models of primary and secondary right ventricular pressure overload (RVPO). A) Peak systolic pressure, B) End-diastolic pressure, C) Heart rate, D) End-diastolic volume, E) End-systolic volume, F) Stroke volume, G) dP/dt max, H) Ventricular stroke work, and I) Cardiac output. *, p<0.05 vs Sham for the corresponding ventricle; †, p<0.05 vs Primary RVPO for the corresponding ventricle; ‡, p<0.05 vs the RV for the same RVPO condition.

To explore the impact of primary and secondary RVPO on biventricular function, steady-state hemodynamic analysis was performed using conductance catheterization in closed-chest, non-invasively ventilated mice (Figure 1). In sham controls, baseline RV peak systolic pressure, dP/dtmax, and stroke work were significantly lower than LV indices (Figure 2 and Table S1). No significant difference in RV pressure was observed after 7 days of secondary RVPO (Figure S1A). Compared to sham-controls, peak RV systolic pressure was increased with no change in RV end-diastolic pressure in both 7-day primary and 10-week secondary RVPO. RV end-diastolic volume was significantly increased in 7-day primary RVPO only, while end-systolic volume was increased in both 7-day primary and 10-week secondary RVPO compared to sham controls. As a result, compared to sham controls, RV ejection fraction was reduced in both 7-day primary (57+15% vs 10+4%, sham vs RV, p<0.01) and 10-week secondary (59+11% vs 25+12%, sham vs RV, p<0.01) RVPO. Compared to sham controls, RV-dP/dtmax was increased in 7-day primary RVPO, but was unchanged in 10-week secondary RVPO. Both RV stroke work and cardiac output were decreased in 7-day primary and 10-week secondary RVPO groups.

Compared to sham controls and 7-day primary RVPO, peak LV systolic pressure was increased in the 10-week secondary RVPO group (Figure 2 and Table S2). In contrast to the RV, LV end-diastolic pressure, end-diastolic volume, and end-systolic volume were unchanged in the 7-day primary RVPO group compared to controls, but all three indices were increased in the 10-week secondary RVPO group. Compared to sham controls, LV dP/dt_max_ was decreased in 10-week secondary RVPO only but remained higher than RV dP/dtmax in each group. Similarly, LV stroke work was decreased in the 7-day primary and 10-week secondary RVPO compared to sham controls but remained higher than RV stroke work in each group.

### Ventriculo-Arterial Coupling Ratios in Primary and Secondary RVPO

To further study the impact of RVPO on biventricular function, ventriculo-arterial coupling (VAC) ratios of arterial elastance:end-systolic elastance (Ea:Ees) were measured for each ventricle. The ratio of RV-VAC to LV-VAC was defined as the BiV-VAC ratio (Table 1). In the 7-day primary RVPO group, RV-Ea was increased and RV-Ees was unchanged, while both LV-Ea and LV-Ees were unchanged compared to sham controls. As a result, RV-VAC was increased and LV-VAC was unchanged, thereby leading to an increased BiV-VAC ratio compared to sham controls. In 10-week secondary RVPO, both RV-Ea and LV-Ea were increased, while RV-Ees remained unchanged and LV-Ees decreased compared to sham controls. As a result, RV-VAC was not significantly changed, while LV-VAC increased, thereby resulting in a reduced BiV-VAC ratio compared to sham-controls.

**Table 1:** Biventricular (BiV) ventriculo-arterial coupling ratios of arterial elastance (Ea) and end-systolic elastance (Ees) in the right (RV) and left ventricles (LV) using models of primary and secondary right ventricular pressure overload (RVPO).

	Sham		Primary RVPO		Secondary RVPO	
	RV	LV	RV	LV	RV	LV
Ea	2.8±1.3*	8.6±3.9	9.9±4.5†	7.5±1.5	7.1±2.2† ^#^	31±12‡
Ees	2.9±2.1*	7.6±4.5	3.4±1.5§	8.4±6.2	3.1±2.1#	4.8±0.9‡
Ea/Ees	1.05±0.5	1.2±0.3	2.4±0.4†	0.8±0.3	1.8±0.8#	5.8±1.7‡
BiV Ratio	0.87±0.3		1.5±0.19**		0.33±0.07**	

* p<0.05: Sham RV vs Sham LV.

§ p<0.05: Primary RVPO RV vs LV.

† p<0.05: Sham RV vs Primary or Secondary RVPO RV.

# p<0.05:Secondary RVPO RV vs LV.

‡ p<0.05: Sham LV vs Primary or Secondary RVPO LV.

### Distinct Effects of Primary and Secondary RVPO on Ventricular Mass

Compared to sham-operated controls, total body weight was significantly reduced after 7-days of primary RVPO only (Table 2). In the 10-week secondary RVPO group, total body weight was unchanged, while total lung weight was significantly increased. Compared to sham controls, RV mass was increased by a similar magnitude in both the 7-day primary and 10-week secondary RVPO groups. In contrast, LV mass (LV free wall and inter-ventricular septum) was increased after 10-weeks of secondary RVPO, but reduced after 7-days of primary RVPO. No change in RV mass was observed, while LV mass was increased in the 7-day secondary RVPO group (Figure S1B).

**Table 2:** Total body, lung, and right (RV) and left (LV) ventricular weights normalized to tibia length in models of primary and secondary right ventricular pressure overload (RVPO).

	Sham	Primary RVPO	Sham	Secondary RVPO
Total Body Weight (g)	33±4	26±1*	32±4	29±4
Total Lung Weight (mg)	172±20	130±30	180±40	410±120* †
RV Weight/Tibia (mg/mm)	1.5±0.2	2.5±0.5*	1.4±0.2	2.5±0.5*
LV Weight/Tibia (mg/mm)	6±0.4	4±1*	6±1	12±2* †

* p<0.05 vs Sham.

† p<0.05, vs Primary RVPO.

### Hypertrophic Remodeling and Calcineurin Expression in RVPO

**Figure 3:**
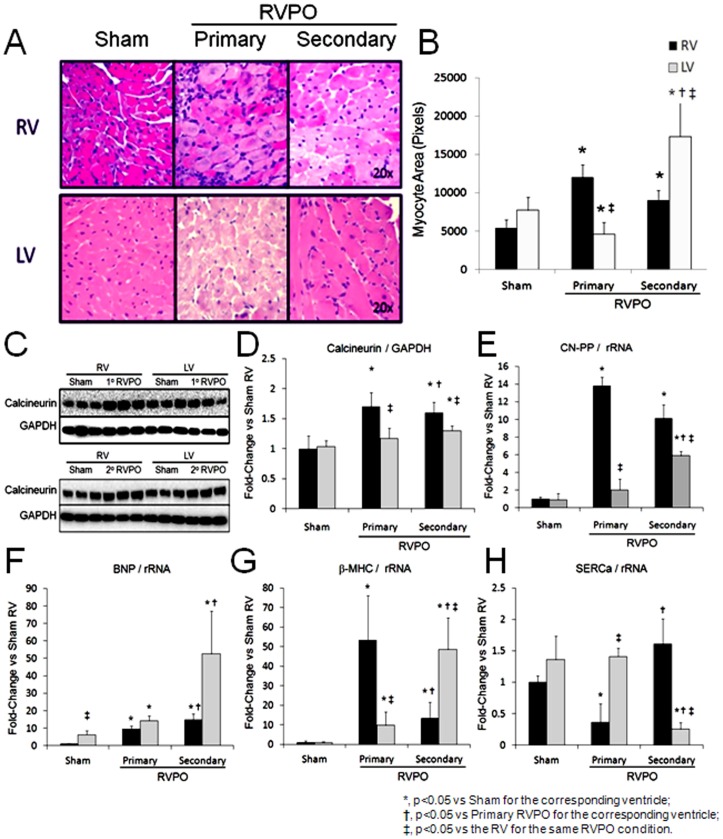
Hypertrophic remodeling in models of primary and secondary right ventricular pressure overload (RVPO). A) Representative histologic staining of right (RV) and left (LV) ventricular tissue and B) bar graph of RV and LV cardiomyocyte cross-sectional areas after primary and secondary RVPO. C) Western blot and D) bar graph of RV and LV calcineurin protein expression normalized to GAPDH. E) Calcineurin-Aβ (CN-PP), F) brain natriuretic peptide (BNP), G) beta-myosin heavy chain (b-MHC), and H) sarcoplasmic reticulum Ca^2+^ATPase (SERCa) gene expression normalized to total ribosomal RNA (rRNA). *, p<0.05 vs Sham for the corresponding ventricle; †, p<0.05 vs Primary RVPO for the corresponding ventricle; ‡, p<0.05 vs the RV for the same RVPO condition.

Compared to sham controls, both 7-days of primary and 10-weeks of secondary RVPO increased RV cardiomyocyte cross-sectional area (Figure 3). LV cardiomyocyte area was decreased in primary RVPO, but increased in secondary RVPO. Consistent with these changes, RV calcineurin protein expression was significantly increased in both models of RVPO, while LV expression was increased in the 10-week secondary RVPO group only. To confirm changes in calcineurin expression, mRNA levels of the calcineurin-A beta-isoform (CN-PP) were measured. RV levels of CN-PP were increased in 7-day primary and 10-week secondary RVPO groups, while LV levels were increased in 10-week secondary RVPO only. We next examined expression of genes regulated by calcineurin that are involved in cardiac hypertrophy. Compared to sham controls, biventricular gene expression of brain natriuretic peptide (BNP) and beta-myosin heavy chain (β-MHC) were increased in both the 7-day primary and 10-week secondary RVPO groups, however in contrast to BNP, levels of β-MHC expression were highest in the RV and LV of 7-day primary and 10-week secondary RVPO groups respectively. RV expression of the sarcoplasmic reticulum Ca^2+^ATPase (SERCa) gene was reduced by 7-day primary RVPO only. LV SERCa gene expression was reduced in the 10-week secondary RVPO group only. No significant increase in calcineurin mRNA expression was observed in the RV after 7-days of secondary RVPO (Figure S1C).

### Fibrotic Remodeling and TGFb1 Expression in RVPO

**Figure 4:**
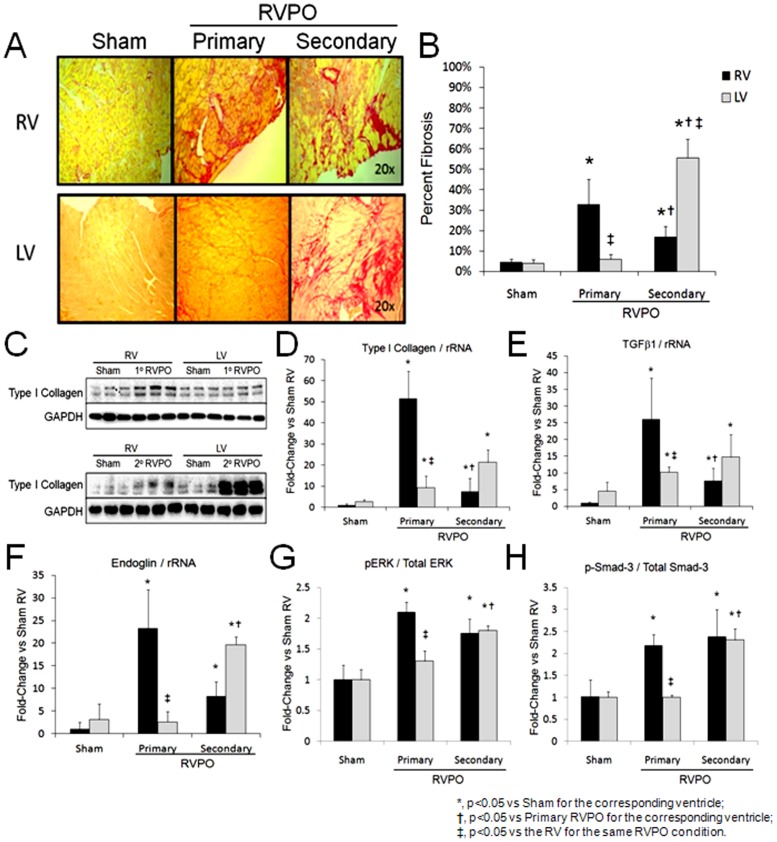
Fibrotic remodeling in models of primary and secondary right ventricular pressure overload (RVPO). A) Picrosirius red staining for collagen abundance and B) quantitation of percent fibrosis in the right (RV) and left ventricle (LV) after primary and secondary RVPO. C) Western blot and D) bar graph of Type I collagen normalized to GAPDH. E–F) Gene expression of transforming growth factor beta 1 (TGFβ1) and endoglin normalized to ribosomal RNA (rRNA). G–H) Quantified protein expression of phosphorylated ERK (pERK) normalized to total ERK and phosphorylated Smad-3 normalized to total Smad-3. *, p<0.05 vs Sham for the corresponding ventricle; †, p<0.05 vs Primary RVPO for the corresponding ventricle; ‡, p<0.05 vs the RV for the same RVPO condition.

Compared to sham controls, both 7-days of primary and 10-weeks of secondary RVPO increased RV collagen deposition and both Type I collagen mRNA and protein expression (Figure 4). Increased LV collagen deposition and Type I collagen protein expression were observed only in the 10-week secondary RVPO group. LV Type I collagen mRNA was increased in both the 7-day primary and 10-week secondary RVPO. TGFβ1 gene expression was increased in both ventricles after 7-days of primary and 10-weeks of secondary RVPO. Levels of the pro-fibrogenic TGFβ1 co-receptor, Endoglin, were increased in the RV after both 7-days of primary and 10-weeks of secondary RVPO and also increased in the LV after 10-weeks of secondary RVPO only along with TGFβ1 signaling via extracellular regulated kinase (ERK) and Smad-3. No significant increase in TGFβ1 mRNA expression was observed in the RV after 7-days of secondary RVPO (Figure S1D).

## Discussion

The impact of RVPO on biventricular structure and function remains poorly understood. We report a percutaneous approach to study pressure volume loops in closed-chest mice and demonstrate distinct biventricular hemodynamic responses to primary and secondary RVPO and further identify increased RV expression of two critical proteins involved in cardiac remodeling, namely calcineurin and TGFβ1.

We demonstrate that biventricular pressure volume analysis via simultaneous cannulation of the internal jugular vein and carotid artery is feasible in murine models of primary and secondary pulmonary hypertension. Despite major advances in murine models of PH and heart failure, invasive hemodynamic studies of biventricular function in these models remains technically challenging and often requires ventricular puncture through the chest wall. Given the increasing importance of transgenic mouse models, the ability to study biventricular hemodynamics may provide new insight into the mechanisms underlying cardiac remodeling. By preserving chest wall dynamics, we observed increased RV volumes with no change in RV filling pressures in both models of RVPO. In contrast, LV pressure and volume were increased in the secondary RVPO group. Furthermore, we show that short-term LV pressure overload does not significantly increased RV pressure in a mouse model of thoracic aortic constriction. These findings indicate that stretch-sensitive signaling pathways may play a central role in remodeling of the thin-walled RV.

To further explore biventricular interactions during RVPO, we studied a well-established marker of uni-ventricular efficiency, namely, the ventriculo-arterial coupling (VAC) ratio in the context of biventricular function. We observed that in both models of RVPO, RV contractile function was recruited to maintain ventriculo-arterial coupling, however with suboptimal efficiency. By measuring ratios of RV-VAC to LV-VAC as an indicator of ’biventricular efficiency’, we first confirmed that the BiV-VAC ratio was approximately 1.0 in sham controls, which is consistent with optimal uni-ventricular efficiency. Surgical constriction of the pulmonary artery and thoracic aorta yielded an expected increase in end-systolic pressure coupled with reduced stroke volume, and thereby resulted in a net increase in arterial elastance (Ea). RV-Ea was similar in both acute, primary and chronic, secondary RVPO. In both models, load-dependent (dP/dtmax) and –independent (Ees) indices of RV contractile function were preserved, while RV ejection fraction was significantly reduced. As a result, distinct BiV-VAC ratios were observed in primary and secondary RVPO. Taken together, these findings suggest that increased afterload alone may not fully account for RV failure associated with pulmonary hypertension or left ventricular failure. Our findings are consistent with studies that have observed a similar degree of ‘RV resilience’ in the setting of pressure and volume overload [31].

We next examined the impact of RVPO on ventricular mass and first observed that total body weight was significantly reduced in primary RVPO, not secondary RVPO. Despite this profound difference in total body weight, RV mass increased to the same degree in both models of RVPO while LV mass was reduced in primary RVPO, but increased in secondary RVPO. Changes in cardiomyocyte cross-sectional area were consistent with changes in ventricular mass. Importantly, seven days of LV pressure overload increased LV mass, but did not affect RV mass, thereby suggesting that RV remodeling is a later consequence of LV pressure overload. A recent clinically study reported a similar pattern of atrophic remodeling of the LV in pulmonary hypertension that may be reversible in conditions such as chronic thromboembolic pulmonary hypertension [32]. One possible explanation for ‘atrophic remodeling of the LV in primary RVPO is the reduction in LV stroke work that occurs with reduced LV preload due to fixed pulmonary vascular obstruction. Future studies are needed to define the cause and significance of LV remodeling in RVPO. Our findings now extend this clinical observation to a preclinical model and further show no significant change in LV contractile function despite reduced LV mass in primary RVPO.

Next, we explored two central pathways that mediate cardiac remodeling, namely, signaling via calcineurin and TGFβ1. Based on numerous studies of left heart failure, calcineurin has been identified as regulator of cardiac hypertrophy, fetal gene expression, and fibrosis [22]–[24]. Few studies have examined calcineurin expression in models of right heart failure [25]. We now show that both primary and secondary RVPO are associated with increased RV calcineurin expression and activity. Furthermore, expression of fetal genes and proteins associated with cardiac hypertrophy followed a typical profile associated with pressure overload with the exception of RV SerCa levels in secondary RVPO, which remained unchanged despite a similar increased in RV pressure and Ea. This difference may reflect the acute versus chronic nature of the two RVPO models or may reflect a functional adaptation by the RV in response to chronic overload and calcium handling.

TGFβ1 is a ubiquitously expressed master switch that induces the fibrotic program in various cell types including cardiac fibroblasts and has been implicated in multiple fibroproliferative diseases including: glomerulosclerosis, ulcerative colitis, hepatic fibrosis, glaucoma, and scleroderma [33]–[37]. To date, preclinical studies have focused on inhibiting TGFβ1 activity in left heart failure by disrupting ligand-receptor binding with modest reductions in cardiac fibrosis [19]–[21]. No studies have examined the role of TGFβ1 signaling in RVPO. We now report that fibrosis is a central aspect of RV remodeling in response to primary or secondary RVPO and further show that TGFβ1 signaling via canonical and non-canonical pathways is upregulated in the RV.

There are several limitations to this study. First, since we used a retrograde approach to the LV and RV from the carotid artery and internal jugular vein, small changes in ventricular volumes may be due to aortic or tricuspid regurgitation. Second, simultaneous recordings of RV and LV loops were not currently feasible due to far-field and near-field interactions from the two conductance catheters despite the use of dual frequency modes. This was resolved by acquiring RV and LV PV loops in rapid sequence during the same setting. Third, cardiac dimensions were not quantified in this study. Finally, future studies employing other time points of RVPO and murine models of primary pulmonary hypertension will be necessary to further define the course of RV remodeling in response to primary and secondary RVPO.

A primary cause of death for individuals with acute or chronic pulmonary hypertension and left heart dysfunction is RV failure which is a directly related to abnormally high pulmonary pressures [1]–[5]. At present, no specific therapies are designed to improve changes in RV structure or function in the setting of primary or secondary pulmonary hypertension. Further studies examining the distinct effects of primary and secondary RVPO on biventricular structure, function, and signaling via the calcineurin and TGFβ1 pathways may identify novel targets of therapy for RV failure.

## Supporting Information

Figure S1 **Biventricular remodeling after 7 days of secondary RVPO due to thoracic aortic constriction (TAC).** A) Compared to sham controls, LV systolic pressure was increased (94+6 vs 132+18 mmHg, Sham vs TAC, p = 0.02) and RV systolic pressure unchanged (23+4 vs 26+3 mmHg, Sham vs TAC, p = NS) after 7 days of thoracic aortic constriction. B) Compared to sham controls, LV mass normalized to tibia length was increased (6+0.4 vs 7+0.1 mg/mm, Sham vs TAC, p = 0.03) and normalized RV mass unchanged (1.5+0.2 vs 1.4+0.1 mg/mm, Sham vs TAC, p = NS) after 7 days of thoracic aortic constriction. C) Calcineurin mRNA expression was not significantly increased in the RV or LV after TAC. D) TGFβ1 mRNA expression was increased in the LV (p = 0.03), not RV after TAC.
(TIF)

Table S1 **Steady State Hemodynamics in a mouse model of primary RVPO (n = 6/group).**
(DOC)

Table S2 **Steady State Hemodynamics in a mouse model of secondary RVPO (n = 6/group).**
(DOC)
