# Supplementary figures and images for: Biventricular Remodeling in Murine Models of Right Ventricular Pressure Overload
Source: PLoS One. 2013 Jul 30;8(7):e70802. doi: 10.1371/journal.pone.0070802 (PMC3728304; doi:10.1371/journal.pone.0070802)

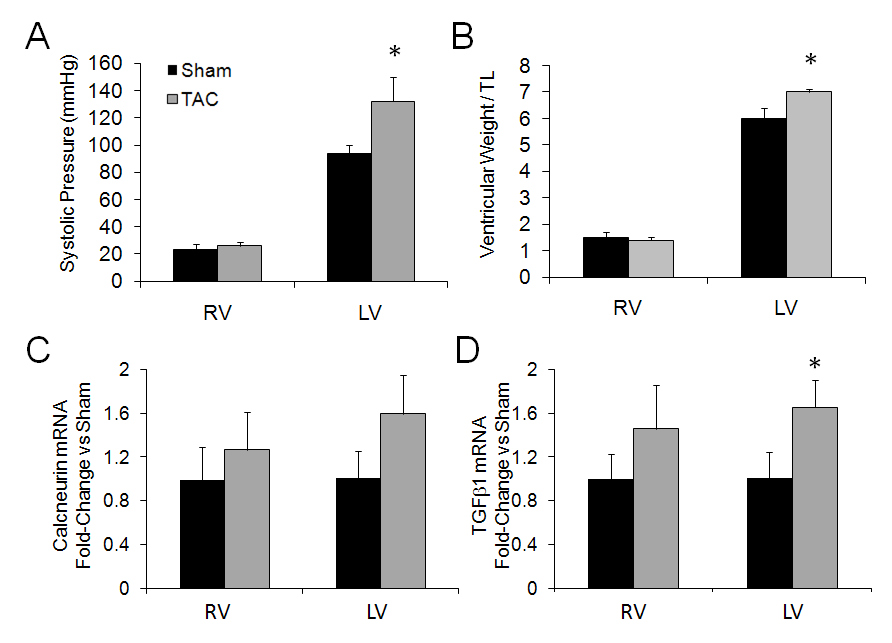

Supplement: Figure S1 — Biventricular remodeling after 7 days of secondary RVPO due to thoracic aortic constriction (TAC). A) Compared to sham controls, LV systolic pressure was increased (94+6 vs 132+18 mmHg, Sham vs TAC, p = 0.02) and RV systolic pressure unchanged (23+4 vs 26+3 mmHg, Sham vs TAC, p = NS) after 7 days of thoracic aortic constriction. B) Compared to sham controls, LV mass normalized to tibia length was increased (6+0.4 vs 7+0.1 mg/mm, Sham vs TAC, p = 0.03) and normalized RV mass unchanged (1.5+0.2 vs 1.4+0.1 mg/mm, Sham vs TAC, p = NS) after 7 days of thoracic aortic constriction. C) Calcineurin mRNA expression was not significantly increased in the RV or LV after TAC. D) TGFβ1 mRNA expression was increased in the LV (p = 0.03), not RV after TAC. (TIF) [file pone.0070802.s001.tif]
