# Supplementary material for: Biventricular Remodeling in Murine Models of Right Ventricular Pressure Overload
Source: PLoS One. 2013 Jul 30;8(7):e70802. doi: 10.1371/journal.pone.0070802 (PMC3728304; doi:10.1371/journal.pone.0070802)
Supplement: Table S1 — Steady State Hemodynamics in a mouse model of primary RVPO (n = 6/group). (DOC) [file pone.0070802.s002.doc]

Supporting Information.

Table S1. Steady State Hemodynamics in a mouse model of primary RVPO (n=6/group).

|  | Right Ventricle | | Left Ventricle | |
| --- | --- | --- | --- | --- |
|  | Sham | Primary RVPO | Sham | Primary RVPO |
| Peak systolic pressure (mmHg) | 23±4 | 48±12 * | 94±6 | 83±17.6 ‡ |
| End diastolic pressure (mmHg) | 3.3±2.6 | 3±4 * | 4.5±2.2 | 10.6±18.5 |
| Max dP/dt (mmHg/sec) | 2445±449 | 3374±1209 | 8666±1597 † | 6466±2060 ‡ |
| Min dP/dt (mmHg/sec) | 2191±227 | 2568±897 | 8009±1654 † | 4824±1622 * ‡ |
| End diastolic volume (uL) | 19.4±9 | 42±11 * | 20±7 | 24±6 |
| End systolic volume (uL) | 8±7 | 38±11 * | 7±4.5 | 13±6 |
| Stroke volume (uL) | 12±3 | 4.5±0.5 * | 13±4 | 5.8±1.5 * |
| Cardiac output (uL/min) | 6036±1965 | 2126±494 * | 7394±2770 | 3713±1045 * ‡ |
| Ejection fraction (%) | 57±15 | 9.8±3.9 * | 69±20 | 31±18 * ‡ |
| Stroke work (mmHg x mL) | 290±185 | 111±58 * | 1060±385 † | 287±133 * ‡ |
| Effective arterial elastance (mmHg/mL) | 2.4±1.6 | 12.7±4.2 * | 7.3±1.6 † | 7.8±2.3 |
| Heart rate (bpm) | 549±82 | 559±48 | 555±88 | 586±46 |
|  |  |  | * p<0.05, Primary RVPO vs Sham | |
|  |  |  | † p<0.05 Sham RV vs Sham LV | |
|  |  |  | ‡,p<0.05, Primary RVPO: RV vs LV | |

Table S2. Steady State Hemodynamics in a mouse model of secondary RVPO (n=6/group).

|  | Right Ventricle | | Left Ventricle | |
| --- | --- | --- | --- | --- |
|  | Sham | Secondary RVPO | Sham | Secondary RVPO |
| Peak systolic pressure (mmHg) | 24+4 | 34.9+5 * | 94+7 † | 107+22 ‡ |
| End diastolic pressure (mmHg) | 2.6+3 | 3.6+1 | 1.9+1 | 19+11 * ‡ |
| Max dP/dt (mmHg/sec) | 2509+557 | 1946+273 | 8885+1706 † | 5246+1260 * ‡ |
| Min dP/dt (mmHg/sec) | 2298+149 | 2147+476 | 8624+1309 † | 4736+1675 * ‡ |
| End diastolic volume (uL) | 19+11 | 20+6 | 18+8 | 31+11 * |
| End systolic volume (uL) | 9.3+8.5 | 15.5+5.6 | 5.9+5.1 | 22.7+10 * |
| Stroke volume (uL) | 10.4+2.2 | 4.9+2.7 * | 11.9+4.2 | 5.3+2.9 * |
| Cardiac output (uL/min) | 5123+1482 | 2783+1588 * | 6764+3226 | 3049+1823 * |
| Ejection fraction (%) | 59+11 | 25+12 * | 74+23 | 27+25 * |
| Stroke work (mmHg x mL) | 196+70 | 113+75 | 983+400 † | 335+263 * ‡ |
| Effective arterial elastance (mmHg/mL) | 2.9+1.8 | 7.6+2.3 * | 7.8+1.7 † | 27.7+16 * ‡ |
| Heart rate (bpm) | 553+94 | 561+23 | 544+111 | 564+34 |
|  |  |  | * p<0.05, Secondary RVPO vs Sham | |
|  |  |  | † p<0.05 Sham RV vs Sham LV | |
|  |  |  | ‡,p<0.05, Secondary RVPO: RV vs LV | |
